# Critical roles of nicotinic acetylcholine receptors in olfactory memory formation and retrieval in crickets

**DOI:** 10.3389/fphys.2024.1345397

**Published:** 2024-02-09

**Authors:** Yukihisa Matsumoto, Chihiro Sato Matsumoto, Makoto Mizunami

**Affiliations:** ^1^ Institute of Education, Liberal Arts and Sciences Division, Tokyo Medical and Dental University, Ichikawa, Chiba, Japan; ^2^ Graduate School of Life Sciences, Tohoku University, Sendai, Miyagi, Japan; ^3^ Faculty of Science, Hokkaido University, Sapporo, Hokkaido, Japan; ^4^ Research Institute for Electronic Science, Hokkaido University, Sapporo, Hokkaido, Japan

**Keywords:** nicotinic acetylcholine receptors, cricket, olfactory learning, long-term memory, nicotine, mecamylamine, methyllycaconitine

## Abstract

Acetylcholine (ACh) is a major excitatory neurotransmitter in the insect central nervous system, and insect neurons express several types of ACh receptors (AChRs). AChRs are classified into two subgroups, muscarinic AChRs and nicotinic AChRs (nAChRs). nAChRs are also divided into two subgroups by sensitivity to α-bungarotoxin (α-BGT). The cricket *Gryllus bimaculatus* is one of the useful insects for studying the molecular mechanisms in olfactory learning and memory. However, the roles of nAChRs in olfactory learning and memory of the cricket are still unknown. In the present study, to investigate whether nAChRs are involved in cricket olfactory learning and memory, we tested the effects of two different AChR antagonists on long-term memory (LTM) formation and retrieval in a behavioral assay. The two AChR antagonists that we used are mecamylamine (MEC), an α-BGT-insensitive nAChR antagonist, and methyllycaconitine (MLA), an α-BGT-sensitive nAChR antagonist. In crickets, multiple-trial olfactory conditioning induced 1-day memory (LTM), whereas single-trial olfactory conditioning induced 1-h memory (mid-term memory, MTM) but not 1-day memory. Crickets injected with MEC 20 min before the retention test at 1 day after the multiple-trial conditioning exhibited no memory retrieval. This indicates that α-BGT-insensitive nAChRs participate in memory retrieval. In addition, crickets injected with MLA before the multiple-trial conditioning exhibited MTM but not LTM, indicating that α-BGT-sensitive nAChRs participate in the formation of LTM. Moreover, injection of nicotine (an nAChR agonist) before the single-trial conditioning induced LTM. Finally, the nitric oxide (NO)-cGMP signaling pathway is known to participate in the formation of LTM in crickets, and we conducted co-injection experiments with an agonist or inhibitor of the nAChR and an activator or inhibitor of the NO-cGMP signaling pathway. The results suggest that nAChR works upstream of the NO-cGMP signaling system in the LTM formation process.

## Introduction

Acetylcholine (ACh) is the most abundant excitatory neurotransmitter in the insect central nervous system (Heinrich et al., 1997; Lee and O’Dowd, 1999; Oleskevitch, 1999; Gauglitz and Pflüger, 2001). Receptors for acetylcholine can be broadly classified into G-protein-coupled muscarinic acetylcholine receptors (mAChRs) and ion-channel nicotinic acetylcholine receptors (nAChRs). The number of nAChRs is larger than the number of mAChRs in the insect brain (Gauthier, 2010). Immunochemical and electrophysiological studies have shown that in the brains of honey bees and fruit flies *Drosophila*, nAChRs are abundant in the antennal lobes, the primary olfactory processing center, and in the mushroom bodies, which receive and integrate multiple sensory and motivational signals (Kreissl and Bicker, 1989; Scheidler et al., 1990; Bicker and Hähnlein, 1994; Bicker and Kreissl, 1994; Gu and O’Dowd, 2006; Barbara et al., 2008). Studies in *Drosophila* revealed that mushroom body intrinsic neurons are cholinergic and nAChRs in mushroom body extrinsic neurons are crucial for aversive memory (Barnstedt et al., 2016). Generally, nAChRs are further divided into two types according to their alpha-bungarotoxin (α-BGT) sensitivity. One type is antagonized by α-BGT (α-BGT-sensitive nAChR) and is also antagonized by methyllycaconitine (MLA) (Thany et al., 2007). The other is a receptor type that is not antagonized by α-BGT (α-BGT-insensitive nAChR) and is antagonized by mecamylamine (MEC) (Courjaret and Lapied, 2001; Thany et al., 2003; Thany et al., 2008). The function of these nAChRs in insect learning and memory has been studied in detail using olfactory and tactile learning protocols in honey bees. Based on results of studies using antagonists of α-BGT-sensitive or non-sensitive nAChRs in honey bees, it has been concluded that α-BGT-sensitive nAChRs are involved in LTM formation and α-BGT-insensitive nAChRs are involved in learning acquisition and retrieval (Gauthier, 2010; Dupuis et al., 2012). However, it is unclear whether this is general among the learning and memory systems of insects. In addition, although there have been many studies in which the effects of agonists of nAChRs on learning and memory in honey bees were examined, the effects of the pesticide neonicotinoids were mainly examined (Fischer et al., 2014; Tison et al., 2019; Mustard et al., 2020). Reports on the effects of nicotine, a classical agonist of nAChRs, on learning and memory are limited (Thany and Gauthier, 2005).

The cricket *Gryllus bimaculatus* has a relatively high olfactory learning capability among insects (Matsumoto and Mizunami, 2002a; Matsumoto and Mizunami, 2004; Matsumoto and Mizunami, 2006; Mizunami et al., 2009; Matsumoto et al., 2013a; Matsumoto, 2022). In addition, this insect has favorable attributes for conducting behavioral pharmacology (Matsumoto et al., 2003; Unoki et al., 2005; Matsumoto et al., 2006; Matsumoto et al., 2009; Matsumoto et al., 2013b; Mizunami et al., 2014; Matsumoto et al., 2018) and molecular biology experiments (Takahashi et al., 2009; Awata et al., 2015; 2016; Kulkarni et al., 2023), thereby positioning it as an emerging model insect to investigate the molecular mechanisms of learning and memory. Crickets offer a convenient means of individual examination of the effects of drugs on various stages in learning and memory processes, including learning acquisition, memory formation, storage, and retrieval, through behavioral pharmacology experiments. It has been demonstrated that olfactory learning memories induced by multiple-trial conditioning in crickets can be categorized into three types according to their sensitivity to anesthesia and protein synthesis dependence: short-term memory (STM), medium-term memory (MTM), and long-term memory (LTM) (Matsumoto and Mizunami, 2000; Matsumoto and Mizunami, 2002b; Matsumoto et al., 2003; Matsumoto et al., 2006; Matsumoto et al., 2012; Matsumoto et al., 2016). STM is anesthesia-sensitive and is observed immediately after training up to 20 min. MTM is unaffected by anesthesia and independent of protein synthesis and it is retained from 20 min to 8 h after training. LTM is protein synthesis-dependent and is observed from 8 h after training. We previously reported that the LTM formation process in crickets can be inhibited or induced by the administration of drugs that regulate NO-cGMP signaling, PKA signaling, and other signaling mechanisms (Matsumoto et al., 2006; Matsumoto et al., 2009; Mizunami et al., 2014; Matsumoto et al., 2018). Based on the results of co-administration experiments of these inhibitors and inducers, we have proposed a model of the signaling pathways involved in memory formation. Briefly, long-term memories are formed by activation of NO-cGMP signaling, which requires multiple training trials. A single trial conditioning is insufficient for this process. The activation of NO-cGMP signaling leads to the activation of downstream PKA-cAMP signaling via CNG channels and CaMKII, resulting in phosphorylation of the transcription factor CREB (Matsumoto et al., 2018). However, it is unclear which biomolecules function upstream of the NO-cGMP signaling system.

In this study we examined how MLA and MEC, which are antagonists of two types of nAChRs (α-BGT-sensitive and α-BGT-insensitive), affect learning and memory in olfactory appetitive learning in crickets. Furthermore, nicotine, an agonist of nAChRs, was shown to induce LTM, and the relationship between nAChRs and the NO-cGMP signaling system in the process of LTM formation was demonstrated.

## Materials and methods

### Insects

Adult male crickets, *Gryllus bimaculatus*, at 1 week after the imaginal molt were used. They were reared in a 12 h:12 h light:dark cycle (photophase: 8:00–20:00) at 27°C ± 2°C and were fed a diet of insect pellets and water *ad libitum*. Three days before the start of the experiment, crickets were individually placed in 100-mL glass beakers and they were fed a diet of insect pellets *ad libitum* but were deprived of drinking water to enhance their motivation to search for water. All experiments were carried out in the photophase.

### Classical olfactory conditioning and testing

In this study, we performed two types of procedures for olfactory conditioning and testing: Responses to odors were evaluated either by odor preference tests in the testing arena or by maxillary-palpi extension responses (MERs). Apple, banana, or peppermint odor was used as a conditioned stimulus (CS) and water was used as an unconditioned stimulus (US).

#### Olfactory appetitive conditioning and odor preference test in the testing arena

Individual animals received single- or multiple-trial conditioning. For multiple-trial conditioning, three sets of CS-US pairing trials were performed with an inter-trial interval of 5 min. A 1-mL hypodermic syringe containing water was used for appetitive conditioning. A small filter paper soaked with apple or banana essence (green apple flavor and banana flavor, both odors are complex odors purchased from Mikoya Kosho, Co., Ltd., Tokyo, Japan) was attached to the needle of the syringe. For odor presentation, the filter paper was placed within 1 cm of the cricket’s head for 3 s to present an odor, and then water was presented to the mouth. After the conditioning trials, the air in the beaker was ventilated. In each experimental group, approximately half of the crickets were trained with apple odor as CS, and the rest were trained with banana odor as CS.

For the memory retention test, each cricket was subjected to odor preference tests in a test apparatus (testing arena) before and at 1 h or 1 day after conditioning by letting them choose between the conditioned odor and control odor (apple odor for crickets conditioned with banana odor and *vice versa*). The apparatus used for the preference test was as described previously (Matsumoto and Mizunami, 2002b). In short, on the floor of the “test chamber” of the apparatus, there were two circular holes that connected the chamber with two of three sources of odor. Each odor source consisted of a cylindrical plastic container containing a filter paper soaked with 3 µL solution of apple essence or banana essence, covered with a fine gauze net. The three containers were mounted on a rotatable holder. Two odor sources could be located simultaneously just below the holes at the “offer position” by rotating the holder.

Before the preference test, an animal was transferred from the beaker to the “waiting chamber” of the apparatus and left for 4 min to become accustomed to the surroundings, and then the door to the test chamber was opened. The test started when the animal entered the test chamber. Two minutes later, the relative positions of the odor sources were switched by rotating the container holder. An odor source was considered to have been visited when the animal probed the net top with its mouth. The time spent visiting each odor source was measured cumulatively. The preference test lasted for 4 min. If the total time of visits of an animal to either source was less than 10 s, we considered that the animal was less motivated to visit odor sources, possibly due to a poor physical condition, and the data were rejected. Such individuals were about 5% of all animals tested. At the end of the training, the sliding door was opened and the animal was gently pushed into the waiting chamber, and then the animal was transferred to a beaker.

#### Olfactory appetitive conditioning and test by MERs

Crickets extend their maxillary palpi and vigorously swing them when water is applied to the antennae, which we refer to as maxillary-palpi extension responses (MERs). In the present study, we performed “MER conditioning” (Matsumoto et al., 2015). Briefly, MER was measured during the olfactory appetitive conditioning described above, using a 1-mL syringe containing water with odorant-soaked filter paper on its needle. In the conditioning, individual animals received five sets of CS-US pairing trials, with an ITI of 5 min. We examined the proportion of crickets that showed MER (%MER) during the 3-s odor presentations in classical olfactory conditioning. Two odorants, apple and peppermint (peppermint essence, complex odor purchased from Mikoya Kosho, Co., Ltd., Tokyo, Japan), were used as CS in a balanced manner, i.e., peppermint was the CS for half of the crickets and apple was the CS for the other half.

For evaluation of retention performance, the rates of MER to the paired odor (CS) and the unpaired odor (novel odor) were compared at 10 min and 1 day after conditioning to assess the specificity of the olfactory memories evoked in the retention tests (Matsumoto et al., 2015). Retention tests were separated by at least 4-min intervals. Half of the crickets first received the CS (ex. peppermint) followed by the novel odor (ex. apple), while the other half received the reversed sequence. Odorant stimulation was identical to that of conditioning trials (4 s), but no US was given. Retention tests were performed at around 10 min (STM) and 1 day (LTM) after the last conditioning trial.

### Pharmacology

For drug injection, a small hole was carefully made on the head cuticle with a needle at the central ocellus at around an hour before injection. Through this hole, each animal was injected with 3 µL cricket saline (Matsumoto et al., 2003) containing a drug into the hemolymph of the head using a 10-µL microsyringe (WPI, Tokyo, Japan) (Matsumoto et al., 2003; Matsumoto et al., 2006; Matsumoto et al., 2009). Mecamylamine (MEC), methyllycaconitine (MLA), (−)-nicotine, Nω-nitro-L-arginine methyl ester (L-NAME), 1H-[1,2,4] oxadiazolo-[4,3-a]quinoxalin-1-one (ODQ), S-nitroso-n-acetyl-penicillamine (SNAP), and 8-Bromoguanosine 3':5′-cyclic monophosphate (8br-cGMP) were purchased from SIGMA (Tokyo, Japan). (±)-(E)-4-Ethyl-2-[(E)-hydroxyimino]-5-nitro-3- hexenamide (NOR3) was purchased from Wako (Tokyo, Japan). ODQ was dissolved in cricket saline containing 0.1% DMSO, and all other drugs were dissolved in cricket saline.

### Data analysis

In the odor preference test in an arena, an odor was considered to have been visited when the cricket probed the odor source with the its mouth or palpi. In odor preference tests in a test apparatus, the relative preference of each animal was determined using the preference index (PI) for rewarded odor, defined as tr/(tr + tnr) × 100, where tr was the time spent exploring the odor associated with reward and tnr was the time spent exploring the odor not associated with reward. Wilcoxon’s (WCX) test was used to compare the odor preferences in different tests of a given animal group. The Mann-Whitney *U* (M-W) test was used to compare odor preferences of two different groups. The Kruskal-Wallis (K-W) test was used to compare odor preferences among three or more groups. We found no significant differences in pre-conditioning odor preferences among different groups of animals (Kruskal-Wallis test, *p* > 0.05). For multiple comparisons, the Holm method was used to adjust the significance level. *p* values of <0.05 were considered statistically significant.

In the MER conditioning procedure, the occurrence of MER to odor presentation was measured during learning acquisition and in retention tests. In all experiments, %MER was calculated as the number of crickets that exhibited MER to the CS divided by the total number of crickets studied x100. A repeated measures ANOVA was used for within-group comparison of %MER during acquisition. In the retention test, McNemar’s test was used for pairwise comparison of %MER between the CS and the novel odor. Fisher’s exact test was used for pairwise comparison of %MER of different groups in each conditioning trial.

## Results

### Effects of nicotinic acetylcholine receptor (nAChR) antagonists on LTM retention

At first, we studied the effects of methyllycaconitine (MLA) and mecamylamine (MEC), antagonists of α-BGT-sensitive nAChRs and α-BGT-insensitive nAChRs, respectively, on 1-day memory retention. Each animal was injected with 3 µL saline containing a drug into the head hemolymph at 20 min before 3-trial appetitive olfactory conditioning. The odor preference was tested before conditioning and 1 day after conditioning, when protein synthesis-dependent LTM can be examined (Matsumoto et al., 2003; Matsumoto et al., 2006).

**FIGURE 1:**
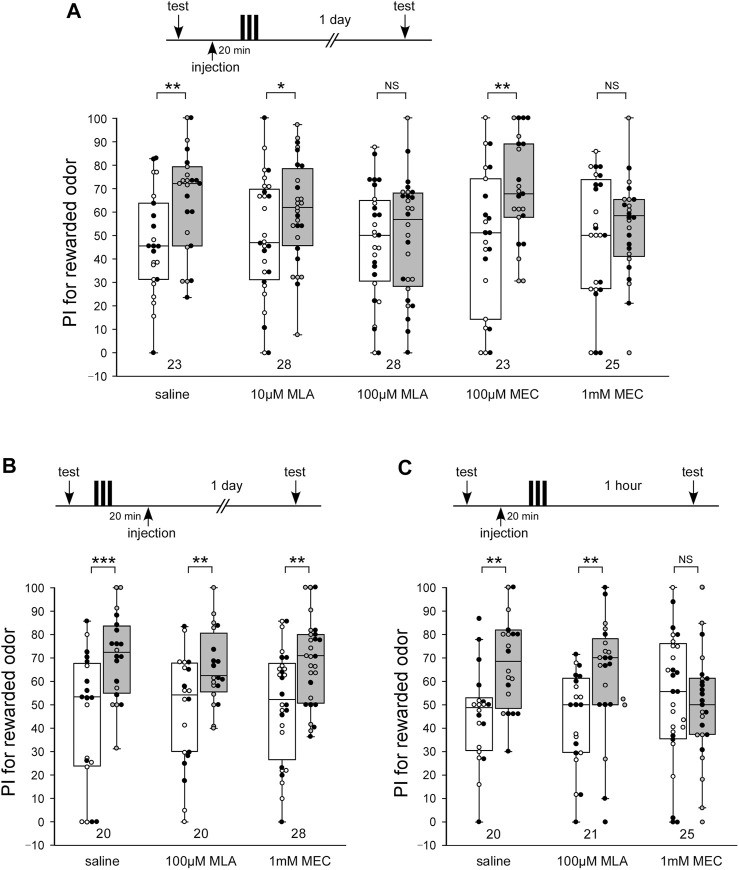
MLA impairs LTM, while MEC impairs both MTM and LTM **(A)** Effects of pre-training application of methyllycaconitine (MLA) and mecamylamine (MEC) on long-term memory (LTM) retention. At 20 min prior to 3-trial conditioning to associate an odor with water reward, crickets in five groups were each injected with 3 µL of saline or saline containing 10 μM MLA, 100 μM MLA, 100 µM MEC or 1 mM MEC. The time schedule of the experiment is shown above the figure. Relative preference between the rewarded odor and control odor was tested before and at 1 day after training. **(B)** Effects of post-training application of MLA and MEC on LTM retention. At 20 min after 3-trial conditioning, crickets in three groups were each injected with 3 µL of saline or saline containing 100 µM MLA or 1 mM MEC. Relative preference between the rewarded odor and control odor was tested before and at 1 day after training. **(C)** Effects of pre-training application of MLA and MEC on medium-term memory (MTM) retention. At 20 min prior to 3-trial conditioning, crickets in three groups were each injected with 3 µL of saline or saline containing 100 µM MLA or 1 mM MEC. Relative preference between the rewarded odor and control odor was tested before and at 1 h after training. Preference indexes (PIs) for the rewarded odor before (white boxes) and after (grey boxes) training are shown as box and whisker diagrams. The individual data was color-coded according to the CS used for conditioning (apple: black dot, banana: open circle). Odor preferences before and after training were compared by the WCX test. The results of statistical comparisons are shown by asterisks (***p < 0.001, **p < 0.01, *p < 0.05, NS *p* > 0.05). The number of animals tested is shown at each data point in this figure and in subsequent figures.

LTM was formed in each animal in the control group that was injected with 3 µL saline 20 min before 3-trial appetitive conditioning: the group exhibited a significantly increased preference for rewarded odor compared with that before conditioning (Figure 1A, WCX test, *p* = 0.00530). In contrast, animals injected with 3 µL saline containing 100 µM MLA or 1 mM MEC 20 min before 3-trial conditioning exhibited no significant level of 1-day retention, indicating that they exhibited no LTM (Figure 1A, WCX test, 100 μM MLA, *p* = 0.3308; 1 mM MEC, *p* = 0.2454). The concentrations of the drugs were determined on the basis of honey bee’s study (Gauthier et al., 2006). No apparent abnormalities were observed in the posture or speed of the crickets after the administration of the MLA or MEC used in this study. The effects of MLA or MEC to LTM were dose-dependent. Animals injected with 10 µM MLA or 100 µM MEC exhibited a significant level of LTM (Figure 1A, WCX test, 10 μM MLA, *p* = 0.0226; 100 µM MEC, *p* = 0.0068), the levels of which were as high as that in the saline-injected group (M-W test, saline vs. 10 μM MLA, *p* = 0.1197; saline vs. 100 µM MEC, *p* = 0.3260). To determine the effective time window of the drug injection, another two groups of crickets were injected with 100 µM MLA or 1 mM MEC at 20 min after the offset of 3-trial conditioning. Both of the drug injection groups exhibited a significant level of LTM (Figure 1B, WCX test, MLA, *p* = 0.00415; MEC, *p* = 0.00730) as in the control group of crickets injected with saline alone 20 min after conditioning (Figure 1B, WCX test, *p* < 0.001). These results suggest that both types of nAChRs participate in any of the memory processes that lead to LTM.

Briefly, the administration of an nAChR antagonist, MLA or MEC, impaired LTM in crickets. To study the effects of MLA and MEC on the earlier memory phase, animals injected with MLA or MEC at 20 min before 3-trial conditioning were tested 1 h after conditioning, which corresponds to amnesia-resistant MTM in crickets (Matsumoto and Mizunami, 2002b; Matsumoto et al., 2003). MLA-injected crickets exhibited a significant level of MTM (Figure 1C, WCX test, *p* = 0.0040), the levels of which were as high as that in the saline-injected group (Figure 1C, M-W test, *p* = 0.3721). In contrast, MEC-injected crickets exhibited no significant level of MTM (Figure 1C, WCX test, *p* = 0.3285). These results indicate that α-BGT-insensitive nAChRs participate in any of the memory processes that lead to MTM, while α-BGT-sensitive nAChRs do not.

### Effects of nAChR antagonists on appetitive conditioning of MER

Administration of MEC prior to training inhibited MTM. Then, would MEC affect the learning acquisition or STM, the memory phase preceding MTM? Although the 4-min olfactory preference test in the arena is suitable for evaluation of memory retention several minutes after training, combining the test with conditioning to evaluate the acquisition of learning was impossible. Olfactory conditioning of MER has an advantage in this point because it allows evaluation of learning acquisition and STM. To study the effects of MLA and MEC on the acquisition of learning or STM, responses to the odors in animals injected with MLA or MEC at 20 min before 3-trial appetitive conditioning were tested during conditioning or at 10 min after conditioning (STM).

**FIGURE 2:**
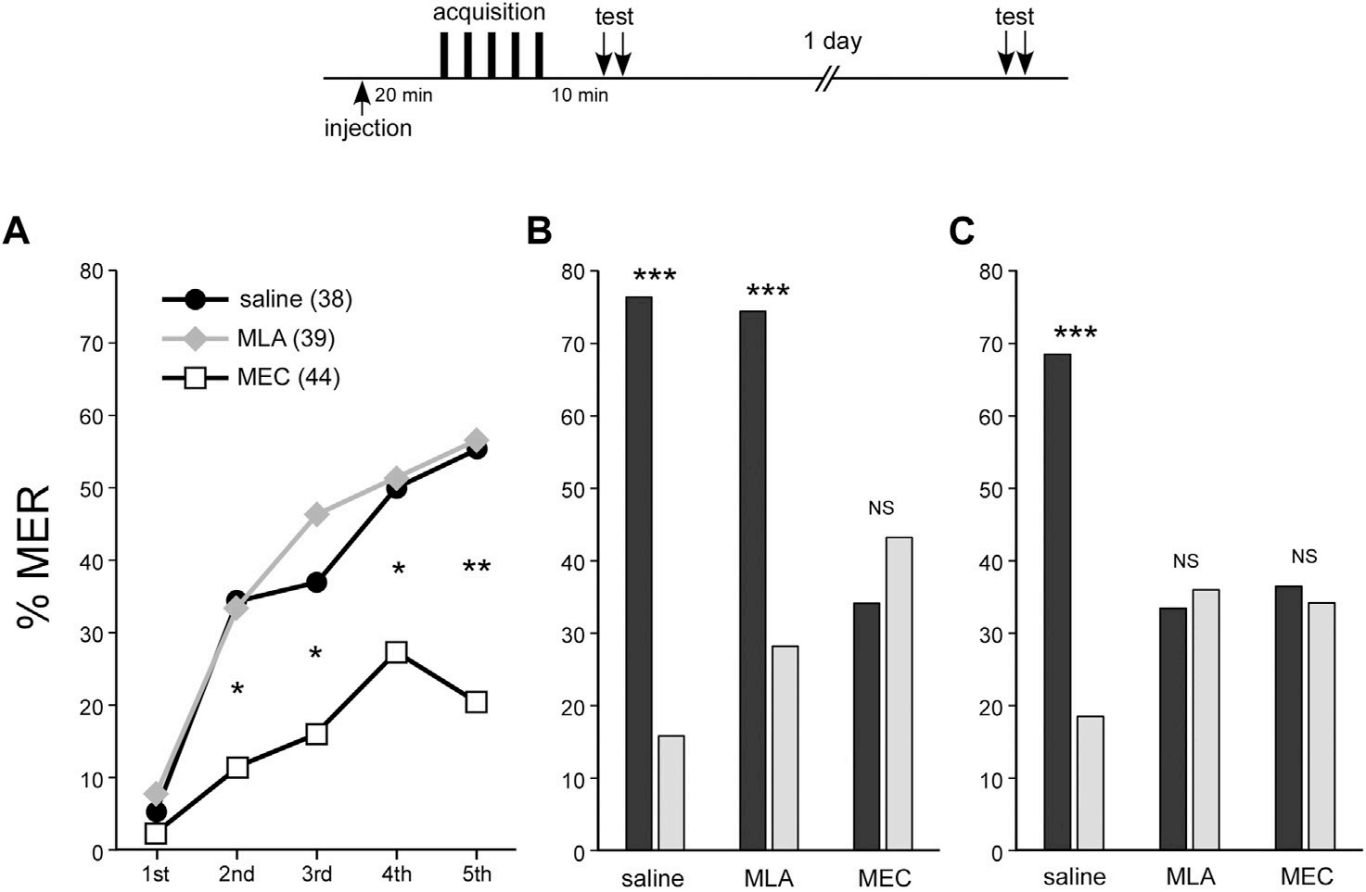
MEC impairs acquisition, STM, and LTM of MER conditioning, while MLA impairs LTM At 20 min before appetitive conditioning, crickets in three groups were each injected with 3 µL of saline (saline group: black circles), saline containing 100 µM MLA (MLA group: gray diamonds) or 1 mM MEC (MEC group: open squares). In all groups, peppermint odor was applied as CS for approximately half of the crickets, and apple odor was applied as CS for the other half. Since the results from these two sub-groups did not significantly differ, datasets were pooled within each group (see Supplementary Figure S2). **(A)** Acquisition performance of appetitive conditioning. The percentage of MER (%MER) during a 3-s period of CS presentation prior to US presentation is shown. **(B)** Retention performance at around 10 min after conditioning. In the retention test, each cricket was tested with the CS and the novel odor separated by a 4-min interval. The saline and MLA groups exhibited a significantly higher %MER to the CS (black bars) than that to the novel odor (gray bars), indicating that the memory is CS-specific. In contrast, in the MEC group, %MER to the CS was as low as that to the novel odor, indicating no CS-specific short-term memory (STM). **(C)** Retention performance at 1 day after conditioning. The saline group exhibited significantly higher %MER to the CS (black bar) than that to the novel odor (gray bar), indicating that the memory is CS-specific. In contrast, in the MLA group and the MEC group, %MER to the CS was as low as that to the novel odor, indicating no CS-specific LTM. A repeated measures ANOVA was used for within-group comparison of %MER during acquisition. McNemar’s test was used for pairwise comparison of %MER between the CS and the novel odor in the retention test. Fisher’s exact test was used for pairwise comparison of %MER of different groups in each conditioning trial. The results of statistical comparisons are shown by asterisks (***p < 0.001, **p < 0.01, *p < 0.05, NS *p* > 0.05).

In the MLA-injected group, %MER to the CS significantly increased with the number of trials (repeated measures ANOVA, *p* < 0.001), demonstrating learning acquisition similar to that in the control group (repeated measures ANOVA, *p* < 0.001) (Figure 2A). In the test 10 min after training, the MLA-injected group showed significantly higher MER (%) to the CS odor compared to the novel odor (McNemar’s test, *p* < 0.001) (Figure 2B). On the other hand, the MEC-treated group also showed significant learning acquisition (repeated measures ANOVA, *p* = 0.0168) (Figure 2A), but in comparison to the saline-injected control group, their scores were significantly lower after the second trial (Fisher’s exact test, first, *p* = 0.4445; second, *p* = 0.0127; third, *p* = 0.0277; fourth, *p* = 0.0293; fifth, *p* = 0.0011). Furthermore, there was no significant difference in MER scores for CS and novel odors at 10 min after training (McNemar’s test, *p* = 0.4227) (Figure 2B). The results imply that associative learning was not established in the MEC-treated group. In an additional memory retention test 1 day after training, MER (%) did not significantly differ between the CS and the novel odor in the MLA- or MEC-injected group (Figure 2C). In other words, both groups exhibited no LTM (McNemar’s test, MLA-injected group, *p* = 0.7055; MEC-injected group, *p* = 0.7389). To add, when crickets that showed MER to the CS but not to the novel odor were defined as crickets with CS-specific memory, the proportion of these crickets at 10 min after training in MEC or MLA injected group was compared with that in the control group (60.5%). The proportion was significantly lower in the MEC group (11.4%) (Fisher’s exact test, *p* < 0.0001), but not in the MLA group (48.7%) (Fisher’s exact test, *p* = 0.2087). One day after training, both the MLA group (10.3%) and the MEC group (9.1%) showed a significant decrease in memory compared to the control group (50.0%) (Fisher’s exact test, *p* < 0.0001).

### Effects of nAChR antagonists on memory retrieval

**FIGURE 3:**
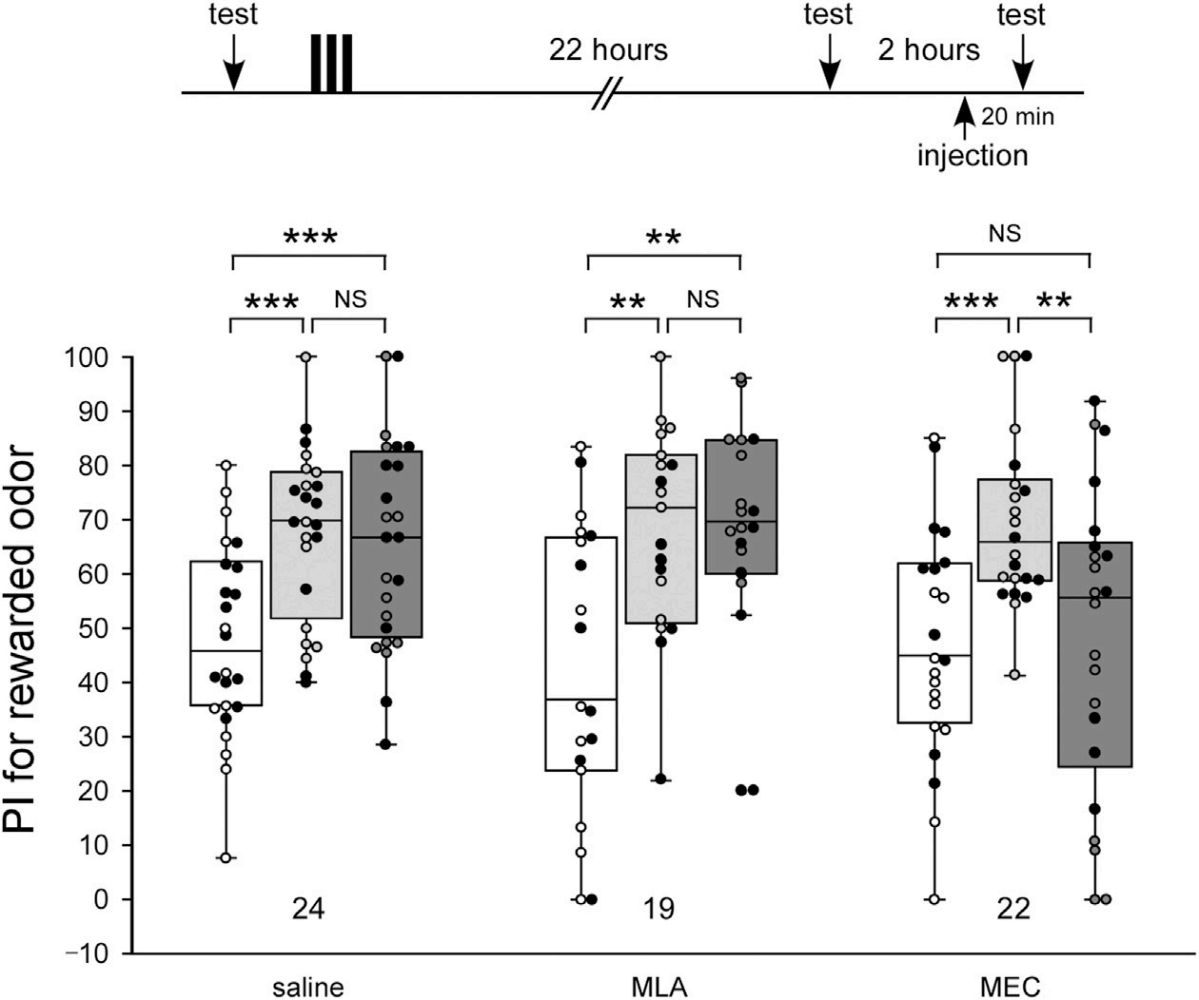
MEC impairs retrieval of LTM Crickets in three groups were each subjected to 3-trial appetitive conditioning. One day after the training, they were each injected with 3 µL of saline or saline containing 100 µM MLA or 1 mM MEC. Relative preference between the rewarded odor and control odor was tested before training (pre-training test), at 22 h after training (before the injection test) and then at 20 min after drug injection (after the injection test). Preference indexes (PIs) for the rewarded odor before training (white boxes), before injection (light gray boxes) and after injection (dark gray boxes) are shown as box and whisker diagrams. The individual data was color-coded according to the CS used for conditioning (apple: black dot, banana: open circle). Odor preferences before and after training were compared by the WCX test. The results of statistical comparisons are shown by asterisks (***p < 0.001, **p < 0.01, NS *p* > 0.05, adjusted by Holm’s method).

To study the effects of nAChR antagonists on memory retrieval, crickets were injected with 100 µM MLA or 1 mM MEC before memory retention tests. In this experiment, odor preference tests were performed 3 times by using the test apparatus: before 3-trial appetitive conditioning (pre-test), 22 h after the conditioning (first retention test), and 24 h after the conditioning (second retention test) (Figure 3). The drugs were injected in crickets 20 min before the second retention test.

In the MLA-injected group, as in the control group injected with saline alone, the level of retention after the drug injection (2nd retention test) was significantly higher than that of the test before conditioning (pre-test) (pre-test vs. 2nd test, WCX test, MLA: *p* = 0.0010, saline: *p* < 0.001) and did not significantly differ from that in the test before the drug injection (first retention test) (1st test vs. 2nd test, WCX test, MLA: *p* = 0.48161, saline: *p* = 0.30166) (Figure 3). In contrast, the MEC-injected group exhibited significantly lower levels of retention after the drug injection (2nd retention test) compared to that before the drug injection (1st retention test) (1st test vs. 2nd test, WCX test, *p* = 0.0049), the levels of which did not significantly differ from that before conditioning (pre-test) (pre-test vs. 2nd test, WCX test, *p* = 0.3074). These results suggest the possibility that α-BGT-insensitive nAChRs, but not α-BGT-sensitive nAChRs, participate in memory retrieval of olfactory learning. Furthermore, when crickets in the MEC-injected group were further tested at 2 h after MEC administration (3rd retention test), they exhibited no significantly different level of retention from that before administration (Supplementary Figure S2, 1st test vs. 3rd test). Thus, the effect of MEC disappeared within 2 h.

To ensure that MEC inhibited odor memory retrieval, it is necessary to exclude the possibility that MEC injection impaired odor reception or discrimination. Therefore, we tested whether MEC inhibits odor discrimination between apple and banana odor. Two groups of crickets were injected with saline (control group) or 1 mM MEC and were tested in the arena before and at 20 min after injection. If MEC administration impairs the odor discrimination between apple and banana odor, the odor preference index for apple odor after injection would get closer to 50. However, this was not the case (Supplementary Figure S3A). Additionally, the injection of MEC did not significantly alter the odor preferences with apple/banana odor pairs compared to the injection of saline (Supplementary Figure S3B). Therefore, we concluded that MEC did not impair odor discrimination in this odor pair.

### Nicotine induces LTM as with activation of the NO-cGMP pathway

In our previous study, we found that 1) multiple-trial conditioning leads to LTM formation but the single-trial conditioning leads to medium-term memory (MTM), 2) injection of inhibitors of key enzymes of the NO-cGMP system or cAMP system prior to multiple-trial conditioning fully impaired LTM formation but had no effect on MTM formation, and 3) injection of an activator of the NO-cGMP system or cAMP system, such as SNAP or 8br-cGMP, prior to single-trial conditioning led to the induction of LTM (Matsumoto et al., 2006; Matsumoto et al., 2009).

**FIGURE 4:**
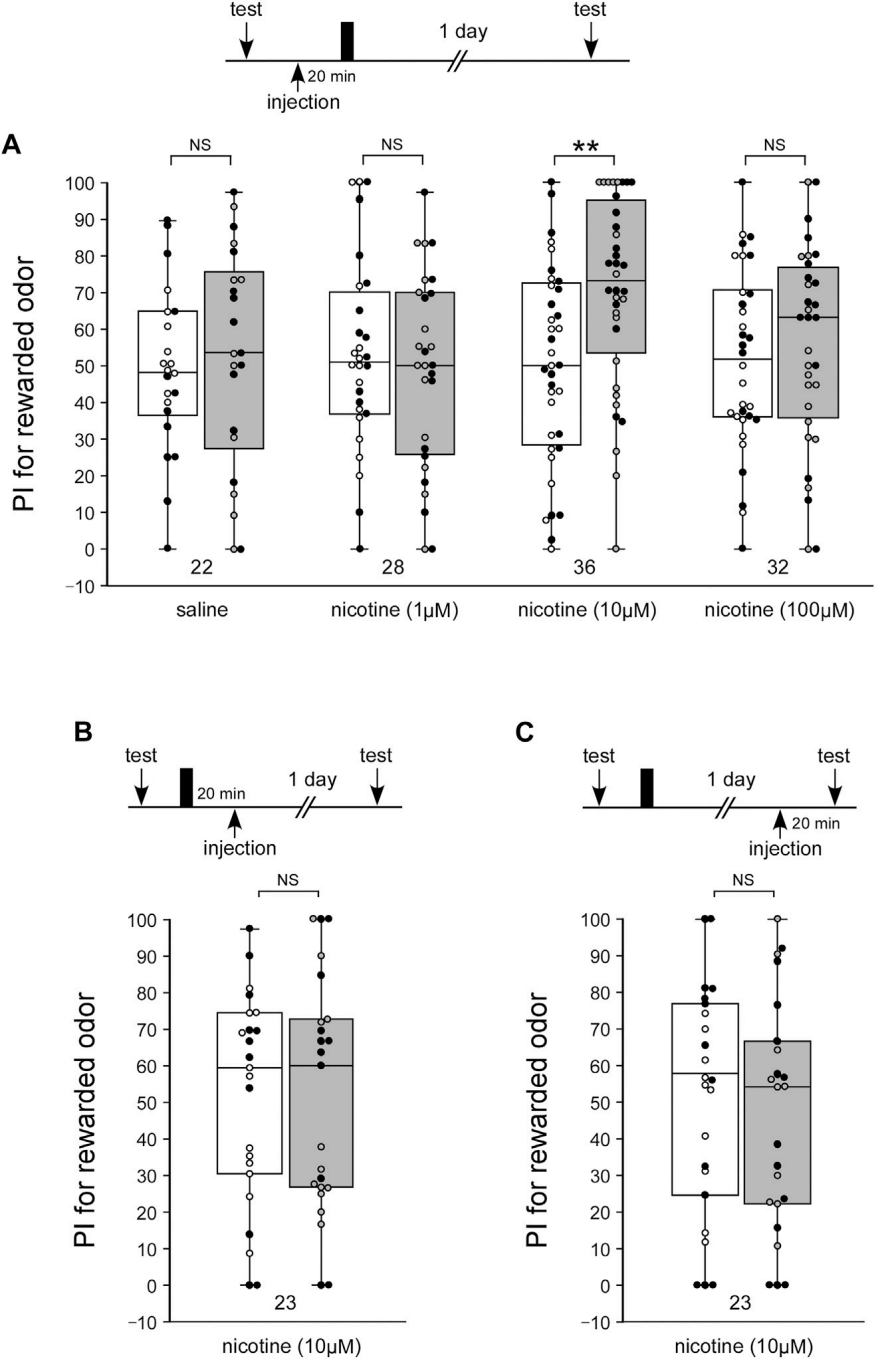
Nicotine application paired with single-trial conditioning induces LTM **(A)** Effects of nicotine injection prior to 1-trial conditioning on LTM formation. At 20 min prior to 1-trial conditioning, crickets in four groups were each injected with 3 µL of saline or saline containing 1 µM nicotine, 10 µM nicotine or 100 µM nicotine. Relative preference between the rewarded odor and control odor was tested before training and at 1 day after training. **(B)** Effects of nicotine injection after 1-trial conditioning on LTM. At 20 min after 1-trial conditioning, crickets were injected with 3 µL of saline containing 10 µM nicotine. **(C)** Effects of nicotine injection prior to the 1-day memory retention test. At 20 min before the retention test 1 day after single-trial conditioning, crickets were injected with 3 µL of saline containing 10 µM nicotine. Preference indexes (PIs) for the rewarded odor before (white boxes) and after (gray boxes) training are shown as box and whisker diagrams. The individual data was color-coded according to the CS used for conditioning (apple: black dot, banana: open circle). Odor preferences before and after training were compared by the WCX test. The results of statistical comparisons are shown by asterisks (**p < 0.01, NS *p* > 0.05).

The behavioral pharmacological experiments in the present study suggest that α-BGT-sensitive nAChRs are involved in LTM formation, while α-BGT-insensitive nAChRs are involved in learning acquisition. Administration of the nAChR agonist nicotine may lead to the induction of LTM as with activators of the NO-cGMP system. To test whether activation of nAChRs can induce of LTM, crickets in four groups were each injected with 3 µL of saline alone or saline containing a different dose of nicotine at 20 min prior to single-trial conditioning. A control group injected with saline alone exhibited no significant level of 1-day retention: the odor preference did not significantly differ from that before conditioning (WCX test, Figure 4A, *p* = 0.3512). The group injected with 1 µM or 100 µM nicotine exhibited no significant levels of 1-day retention (Figure 4A, WCX test, 1 µM: *p* = 0.2986; 100 µM: *p* = 0.2526), whereas the 10 µM-group exhibited a significantly higher level of 1-day retention (Figure 4A, WCX test, *p* = 0.0018). The results suggest that an externally applied activator of nAChRs, as with activators of the NO-cGMP system, can trigger a biochemical cascade leading to LTM formation when paired with single-trial conditioning. We also tested whether nicotine injected before the single-trial conditioning affects MTM. The 1-h retention in the crickets injected with 3 µL of saline containing 10 µM nicotine after single-trial conditioning did not significantly differ from that of the control group (Supplementary Figure S4).

Next, we tested whether nicotine injected after the single-trial conditioning facilitates LTM formation or retrieval. When crickets were injected with 3 µL of saline containing 10 µM nicotine at 20 min after single-trial conditioning, they exhibited no significant level of 1-day retention (Figure 4B, WCX test, *p* = 0.4098). When crickets were injected with the 10 µM nicotine solution at 20 min before the 1-day retention test, no significant level of memory was observed. (Figure 4C, WCX test, *p* = 0.2723). Thus, nicotine injection after single-trial conditioning did not enhance memory formation or retrieval.

### Relationship between nAChRs and the NO-cGMP pathway for induction of LTM

The results showed that nAChRs are required for LTM formation, as is the NO-cGMP pathway. In honey bees, it has been hypothesized that nAChRs function upstream of NO-cGMP signaling in the mechanism of LTM formation (Gauthier, 2010). Then, how are nAChRs and the NO-cGMP pathway sequenced within the LTM formation process in crickets?

**FIGURE 5:**
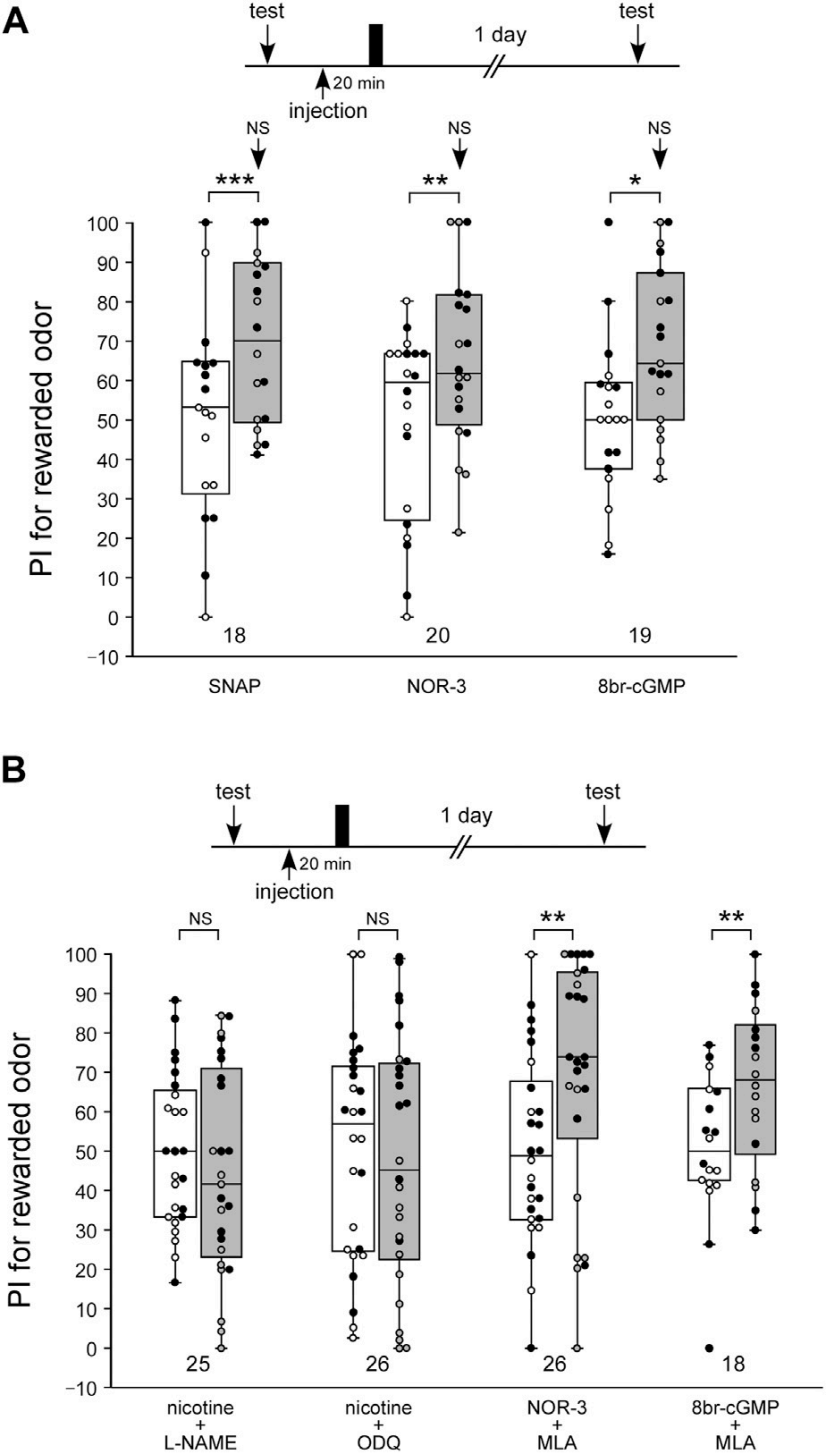
The nAChRs act upstream of the NO-cGMP signaling in the LTM formation process **(A)** Effects of SNAP, NOR-3, and 8br-cGMP paired with 1-trial conditioning on 1-day retention. Crickets in three groups were each injected with 3 µL of saline containing SNAP (200 µM), NOR-3 (40 µM) or 8br-cGMP (200 µM) at 20 min prior to single-trial conditioning. Relative preference between the rewarded odor and control odor was tested before training and at 1 day after training. **(B)** Effects of co-injection of an agonist/antagonist of nAChR and an inhibitor/accelerator of NO-cGMP signaling on 1-day retention. Crickets in four groups were individually co-injected at 20 min before single-trial conditioning with 3 µL of saline containing one of the following pairs of chemicals: nicotine (10 µM) and L-NAME (400 µM), nicotine (10 µM) and ODQ (200 µM), NOR-3 (40 µM) and MLA (100 µM), and 8br-cGMP (200 µM) and MLA (100 µM). Relative preference between the rewarded odor and control odor was tested before and at 1 day after training. PIs for the rewarded odor before (white boxes) and after (grey boxes) training are shown as box and whisker diagrams. The individual data was color-coded according to the CS used for conditioning (apple: black dot, banana: open circle). Odor preferences before and after training were compared by the WCX test. The arrows in the graph **(A)** indicate the results of comparison by the M-W test, between that group and the saline group in Figure 1A, at 1 day after training. The results of statistical comparisons are shown by asterisks (***p < 0.001, **p < 0.01, *p < 0.05, NS *p* > 0.05).

We confirmed our previous findings that activation of the NO-cGMP system led to the formation of LTM (Figure 5A). In this experiment, three groups of animals were each injected with 3 µL of saline containing 200 µM of the NO-donor SNAP, 40 µM of another NO-donor, NOR-3, or 200 µM of the cGMP analog 8br-cGMP at 20 min prior to single-trial appetitive conditioning. The concentrations of the drugs were determined on the basis of our previous study (Matsumoto et al., 2006; Matsumoto et al., 2009). The groups injected with SNAP, NOR-3, and 8br-cGMP before single-trial conditioning exhibited a significant level of LTM: the preference for the rewarded odor was significantly greater than that before conditioning in these groups (Figure 5A, WCX test, SNAP, *p* < 0.001; NOR-3, *p* = 0.0047; 8br-cGMP, *p* = 0.0134). The level of 1-day retention in these groups did not significantly differ from that in the saline-injected control group subjected to multiple-trial conditioning (M-W test, control: Figure 1A saline-injection group; vs. SNAP, *p* = 0.2771; vs. NOR-3, *p* = 0.4709; vs. 8br-cGMP, *p* = 0.3664), thus indicating that the effect of SNAP, NOR-3, or 8br-cGMP to induce LTM is saturated.

Next, we investigated whether induction of LTM by nicotine paired with single-trial conditioning is mediated by the NO-cGMP signaling pathway. NO-cGMP signaling includes the enzyme that synthesizes NO (NO synthase: NOS) and the enzyme that is activated by NO to synthesize cGMP (soluble guanylyl cyclase: sGC). In this experiment, we used L-NAME as the NOS inhibitor and ODQ as the sGC inhibitor. Animals in two groups were each co-injected with nicotine (10 µM) and either the NOS inhibitor L-NAME (400 µM) or the sGC inhibitor ODQ (200 µM), and then the animals were subjected to single-trial conditioning. The concentrations of the drugs were determined on the basis of our previous study (Matsumoto et al., 2006). Neither group exhibited significant differences in the odor preference before and at 1 day after training (Figure 5B, WCX test, nicotine + L-NAME, *p* = 0.1837; nicotine + ODQ, *p* = 0.4111). Thus, LTM was not formed in either group. These findings suggest that induction of LTM facilitated by nicotine with single-trial conditioning was blocked by co-injection of the NOS inhibitor or the sGC inhibitor. Animals in another two groups were each co-injected with the nAChRs inhibitor MLA (200 µM) and either the NO-donor NOR-3 (40 µM) or the cGMP analog 8br-cGMP (200 µM), and then they were subjected to single-trial conditioning. Both groups showed a significant increase in the preference for the CS at 1 day after training compared to that before training (Figure 5B, WCX test, NOR-3 + MLA, *p* = 0.00230; 8br-cGMP + MLA, *p* = 0.00284). Thus, both groups exhibited LTM. The results suggest that induction of LTM by nicotine is mediated by the NO-cGMP pathway, whereas induction of LTM by the NO-cAMP pathway is not mediated by nAChRs.

## Discussion

In this study, we investigated the effects of methyllycaconitine (MLA) and mecamylamine (MEC), antagonists of α-BGT-sensitive and -insensitive nAChRs, respectively, on learning and memory in olfactory appetitive learning in crickets. The results suggested that α-BGT-sensitive nAChRs are critical for LTM formation and that α-BGT-insensitive nAChRs are critical for learning acquisition and memory retrieval. Furthermore, it was shown that nicotine, an agonist of nAChRs, facilitates LTM formation and that α-BGT-sensitive nAChRs function upstream of the NO-cGMP signaling pathway for LTM formation.

### Role of α-BGT-insensitive nAChRs in learning and memory

The effects of MEC on learning acquisition and memory retention were examined. Administration of MEC 30 min before an LTM retention test resulted in a decrease of 1-day olfactory memory scores. There are three possible mechanisms by which MEC may inhibit this 1-day memory: 1) inhibition of odor detection, 2) inhibition of odor discrimination, and 3) inhibition of memory retrieval. In the present study, crickets treated with MEC searched for odors in the arena test and their learning acquisition curves were above zero level in the MER-based tests. This indicates that their olfactory detection ability was preserved despite the effect of MEC. On the other hand, in the odor discrimination test between apple and banana odors, the MEC-treated group did not significantly alter the odor preferences with apple/banana odor pairs compared to the injection of saline. In other words, MEC did not seem to affect odor discrimination. Similar results have been obtained in honey bees using a Y-tube olfactometer, which showed that MEC did not affect odor discrimination (Lozano et al., 1996). Based on these results, we conclude that MEC inhibited memory retrieval.

In the MER conditioning experiments, crickets that received MEC injection before training showed a significant but low level of learning acquisition. Moreover, CS-specific memory (STM) was not observed in the retention test 10 min after training. The significant level of acquisition in MER conditioning may be due to non-associative learning (e.g., increased olfactory sensitivity to odors in general, responding even to odors other than CS). The crickets that received the same MEC treatment before training also showed inhibition of memory in the longer term [1-h retention (MTM) and 1-day retention (LTM)] by arena preference tests, which is consistent with the results of MER-based experiments. Among these results, decreases in the learning acquisition and STM scores could be caused by impairment in memory retrieval by MEC injection before training. However, CS-specific LTM was not observed at 1 day after MEC injection, when the effect of MEC had already disappeared. These results suggest that “MEC injection did not impair CS-US association during training, but impaired LTM formation” or “MEC injection impaired CS-US association during training.” On the other hand, administration of MEC 20 min after training did not inhibit LTM, suggesting that MEC does not affect the mechanism to maintain memory after the establishment of associative learning. The simplest explanation for these results is that MEC injection before training led to the failure of the CS-US association during training. Based on these findings, we conclude that α-BGT-insensitive nAChRs are involved in memory retrieval and CS-US association in olfactory learning.

### Role of α-BGT-sensitive nAChRs in learning and memory

In the present study, pre-training administration of MLA inhibited 1-day retention (LTM) when tested either in the arena or by MER. On the other hand, pre-training administration of MLA did not significantly affect learning acquisition, 10-min retention (STM), or 1-h retention (MTM). It can be said that MLA did not impair odor discrimination or CS-US association. Thus, we conclude that α-BGT-sensitive nAChRs are specifically involved in LTM formation. Furthermore, MLA administration 20 min after training had no inhibitory effect on LTM. This is consistent with the timing of the effects of inhibitors of the NO-cGMP and cAMP-PKA signaling pathways on LTM in our previous study (Matsumoto et al., 2006).

The results of the present study suggest that activation of α-BGT-sensitive nAChRs during or immediately after training is crucial for LTM formation, similar to the signaling molecules involved in the NO-cGMP and cAMP-PKA pathways. Furthermore, unlike MEC, MLA did not inhibit LTM retrieval. These results are consistent with the conclusion from learning experiments using the olfactory and tactile senses of honey bees that α-BGT-sensitive nAChRs are involved in LTM formation (Dacher et al., 2005; Gauthier et al., 2006; Dacher and Gauthier, 2008).

A notable difference was found between crickets and honey bees in the time window of administration of α-BGT-sensitive nAChR antagonists for inhibiting LTM formation. In honey bees, post-training administration of α-BGT or MLA has been shown to inhibit LTM (Gauthier et al., 2006), whereas in crickets, post-training administration of MLA did not inhibit LTM. The activation timing of signaling pathways including α-BGT-sensitive nAChR and NO-cGMP signaling during LTM formation may differ between crickets and honey bees.

### Effects of nicotine on learning and memory

The effect of nicotine, an nAChR agonist, on olfactory memory was examined in this study. Administration of nicotine prior to single-trial conditioning, which typically fails to form LTM, resulted in successful formation of LTM. Nicotine activates both α-BGT-sensitive and α-BGT insensitive nAChRs, but the effect of nicotine to induce LTM is likely to be due to activation of the α-BGT-sensitive form, as MLA inhibits LTM formation. On the other hand, nicotine did not induce LTM when administered 20 min after training. This is consistent with the administration timing of MLA, an α-BGT-sensitive nAChR antagonist that exhibited an inhibitory effect on LTM formation. Based on the results, we conclude that the activity of α-BGT-sensitive nAChRs either during or immediately after training produces the necessary conditions for LTM formation.

A substantial number of reports support the facilitative effects of nicotine on learning and memory in vertebrates including humans (humans: McClernon et al., 2003; Newhouse et al., 2004, rats: Levin, 2002; Puma et al., 1999, mice: Ciamei et al., 2001; Young et al., 2004, rabbits: Woodruff-Pak, 2003; Woodruff-Pak et al., 2000, zebrafish: Levin and Chen, 2004). It has been reported that while nicotine enhances LTM, it does not affect STM (Gould et al., 2014) and that nicotine elongates the duration of memory storage (Lima et al., 2013). In insects, findings on the effects of nicotine on learning and memory are limited. Nicotine has been reported to enhance memory retrieval in single-trial conditioning in honey bees (Thany and Gauthier, 2005) and to enhance memory for floral traits in bumblebees (Baracchi et al., 2017). The present study is the first study to demonstrate that nicotine has a facilitative effect on LTM in insect learning.

In insects, many nAChR agonists other than nicotine (such as neonicotinoid: clothianidin, imidacloprid, thiacloprid, thiamethoxam, dinotefuran; sulfoximine: sulfoxaflor; butanolide: flupyradifurone) are applied as pesticides (Casida, 2018). The effects of these pesticides have attracted interest in the context of sublethal biological effects on beneficial insects, especially honey bees, and learning and memory is one of the extensively documented fields (Fischer et al., 2014; Hesselbach and Scheiner, 2018; Tison et al., 2019; Mustard et al., 2020; Tasman et al., 2021; Cartereau et al., 2022). Contrary to the effects of nicotine in the present study, all of the pesticidal nAChR agonists showed inhibitory effects on learning and memory in honey bees. The chemical doses used in those studies, for example, clothianidin (7.5 ng/bee), imidacloprid (11.25 ng/bee), thiacloprid (1.25 µg/bee) (Fischer et al., 2014), sulfoxaflor (15 ng/bee) (Cartereau et al., 2022), and flupyradifurone (1.2 µg/bee) (Hesselbach and Scheiner, 2018), were sublethal doses for honey bees. When compared by the concentration per body weight (g/kg bw) (estimated body weight: 100 mg/bee, 600 mg/cricket), the effective drug concentrations in most of the honey bee experiments (75 μg/kg–12,500 μg/kg bw) were higher than the effective nicotine concentration in crickets in the present study (8 μg/kg bw, 10 μM nicotine). One exception was the clothianidin experiment (8 μg/kg bw) by Tison et al. (2019), in which a concentration similar to that used in crickets in this study was used. The different effects of nAChRs in crickets and honey bees may be due to differences in the concentration and properties of the chemicals used. In the present study, three concentrations of nicotine (3 µL each, 1 µM: 0.8 μg/kg bw, 10 µM: 8 μg/kg bw, and 100 µM: 80 μg/kg bw) were administered and only the medium concentration (8 μg/kg bw) showed an enhancement effect on LTM formation. While the 10 µM nicotine group showed successful LTM formation, the 100 µM group did not. To explain this difference, we hypothesized that the results of 100 µM nicotine might be due to the overstimulation of nAChRs by higher concentrations of nicotine, which may have led to the failure of basic memory formation process. Sublethal, high-dosage (400 μg/kg bw) nicotine administration inhibited LTM formation with 3 trials of training (data not shown). Thus, nicotine facilitates or inhibits memory depending on the experimental dose in crickets. Similarly, neonicotinoids and other nAChR agonists may facilitate learning and memory at lower concentrations in honey bees. Concentration-dependent effects of these chemicals should also be examined in the olfactory learning system of crickets and other insects.

Nicotine has been reported to participate in age-related memory impairment in aged animals and to ameliorate Alzheimer’s disease and other diseases in vertebrates (Decker et al., 1992; Levin, 1992; Levin and Rezvani, 2002; White and Levin, 2004; Levin et al., 2006). In crickets, age-related memory impairment has been observed in LTM, and such impairment could be rescued by the administration of chemical inducers that function within the LTM formation cascade (Matsumoto et al., 2016). For example, injection of an NO donor that activates the NO-cGMP pathway improves LTM formation in aged crickets. It remains to be determined whether nicotine also improves age-related memory impairment in crickets.

### The α-BGT-sensitive nAChRs act upstream of the NO-cGMP signaling in the biochemical pathways for LTM formation

Behavioral pharmacology experiments using co-administration of inhibitors and inducers of LTM suggested that α-BGT-sensitive nAChRs act upstream of the NO-cGMP signaling in the mechanism of LTM formation in crickets. Administration of nicotine or acetylcholine in the house cricket (*Acheta domesticus*) induced intracellular calcium influx via α-BGT-sensitive nAChRs in Kenyon cells located within the mushroom bodies of the brain (Cayre et al., 1999). Similar observations have been reported in honey bees (Bicker, 1996) and in *Drosophila melanogaster* (Yu et al., 2003). Additionally, intracellular calcium ions activate NO synthase (NOS) (Müller and Bicker, 1994; Müller, 1996). *In vitro* experiments in tobacco hornworms have also shown that the activity of α-BGT-sensitive nAChRs leads to NO synthesis (Zayas et al., 2002). Behavioral pharmacology experiments in crickets and honey bees suggest that NO-cGMP signaling activated by olfactory conditioning triggers several signaling pathways including PKA-cAMP signaling, resulting in the formation of LTM via the transcription factor CREB (Matsumoto et al., 2006; Matsumoto et al., 2018). LTM is formed by spaced multiple-trial conditioning but not by single-trial conditioning. Repetitive training is likely to activate α-BGT-sensitive nAChRs, and the subsequent induction of an intracellular cascade involving NO-cGMP signaling may lead to the formation of LTM.

Various signaling pathways are known to be involved in the formation of LTM in animals other than insects, including NO-cGMP signaling and cAMP-PKA-CREB signaling as well as NMDA-type glutamate receptor signaling (Giese et al., 2015; Wang and Peng, 2016), insulin signaling (Zhao and Alkon, 2001; Dou et al., 2005), mTOR signaling (Bekinschtein et al., 2007; Hylin et al., 2018), and MAPK signaling (Ota et al., 2008; Alfieri et al., 2011). Several inhibitors of these signaling pathways have also been found to inhibit LTM formation for olfactory learning in crickets (Matsumoto, personal communication). Co-administration of the inhibitor and the inducer known to function in LTM formation would further reveal the interrelationships among the signaling systems.

### Future perspectives

The present study suggests that α-BGT-insensitive nAChRs are involved in learning acquisition and memory retrieval, whereas α-BGT-sensitive nAChRs are involved in LTM formation in crickets. One of our future goals is to determine the specific regions of the brain in which these receptors function. Local drug administration experiments used in cockroaches and honey bees may be instructive (Hammer and Menzel, 1998; Menzel and Giurfa, 2006; Watanabe et al., 2011; Matsumoto et al., 2023). In cockroaches, local administration experiments of MEC using salivary gland conditioning suggest that α-BGT-insensitive nAChRs in the calyx or lobes of the mushroom bodies, but not in the antennal lobes or lateral lobes, are involved in CS-US association of olfactory learning (Watanabe et al., 2011). We aim to further examine in crickets whether local administration of MEC or MLA into specific brain regions (e.g., the calyx or lobes of the mushroom bodies, the antennal lobes) inhibits learning acquisition or LTM in order to locate the specific brain regions that are critical for these processes.

## Data Availability

The raw data supporting the conclusion of this article will be made available by the authors, without undue reservation.
