# Supplementary material for: Critical roles of nicotinic acetylcholine receptors in olfactory memory formation and retrieval in crickets
Source: Front Physiol. 2024 Feb 9;15:1345397. doi: 10.3389/fphys.2024.1345397 (PMC10884312; doi:10.3389/fphys.2024.1345397)
Supplement: Supplementary file 1 [file Image4.pdf]

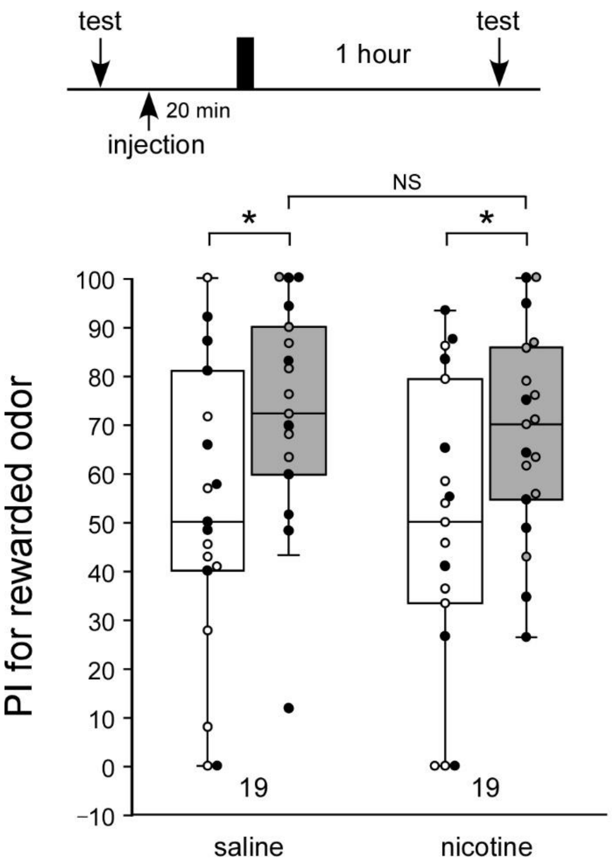

Supplementary Figure S4. Nicotine application paired with single-trial conditioning does not affect MTM

Effects of nicotine injection prior to 1-trial conditioning on MTM formation. At 20 min prior to 1-trial conditioning, crickets in two groups were each injected with 3 μL of saline or saline containing 10 μM nicotine. Relative preference between the rewarded odor and control odor was tested before training and at 1 hour after training. Preference indexes (PIs) for the rewarded odor before (white boxes) and after (gray boxes) training are shown as box and whisker diagrams. The individual data was color-coded according to the CS used for conditioning (apple: black dot, banana: open circle). Odor preferences before and after training were compared by the WCX test. Odor preferences after training of different groups were compared by the M-W test. The results of statistical comparisons

are shown by asterisks (* $P<0.05$, NS $P>0.05$). There was no significant difference in MTM between the nicotine-injected group and the control group (M-W test, $P= 0.5787$). These results suggest that nicotine does not affect MTM.
