# Supplementary material for: Critical roles of nicotinic acetylcholine receptors in olfactory memory formation and retrieval in crickets
Source: Front Physiol. 2024 Feb 9;15:1345397. doi: 10.3389/fphys.2024.1345397 (PMC10884312; doi:10.3389/fphys.2024.1345397)
Supplement: Supplementary file 2 [file Image2.pdf]

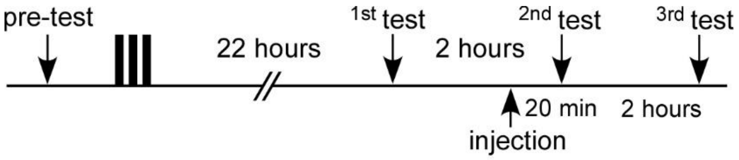

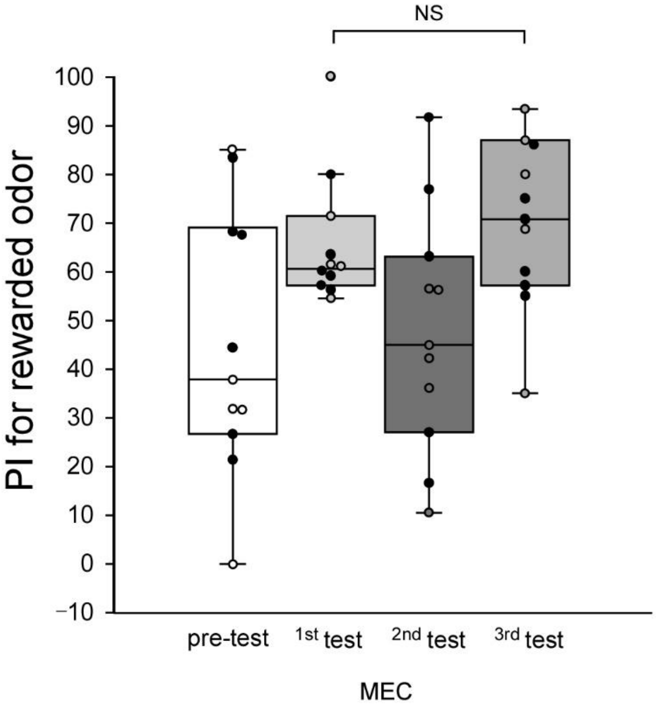

Supplementary Figure S2. Effects of MEC on olfactory memory retrieval disappear within 2 hours

This graph shows a partial dataset in Fig. 3 for the MEC injected group with an additional test (3rd test) that was performed at 2 hours after MEC injection (n=11). Crickets were subjected to 3-trial appetitive conditioning. One day after the training, they were injected with 3 μL of saline containing 1 mM MEC. Relative preference between the rewarded odor and control odor was tested before training (pre-test), at 22 hours after training (1st test) then at 20 min after drug injection (2nd test), and at 2 hours after drug injection (3rd test). Preference indexes (PIs) for the rewarded odor before training (white box), before injection (light gray box) 20 min after injection (dark gray box) and 2 hours after injection

(medium gray box) are shown as box and whisker diagrams. The individual data was color-coded according to the CS used for conditioning (apple: black dot, banana: open circle). Odor preferences did not significantly differ between the 1st test (before MEC injection) and the 3rd test (2 hours after MEC injection) when compared by the WCX test (P=0.3125, NS).
