# Supplementary material for: Critical roles of nicotinic acetylcholine receptors in olfactory memory formation and retrieval in crickets
Source: Front Physiol. 2024 Feb 9;15:1345397. doi: 10.3389/fphys.2024.1345397 (PMC10884312; doi:10.3389/fphys.2024.1345397)
Supplement: Supplementary file 3 [file Image3.pdf]

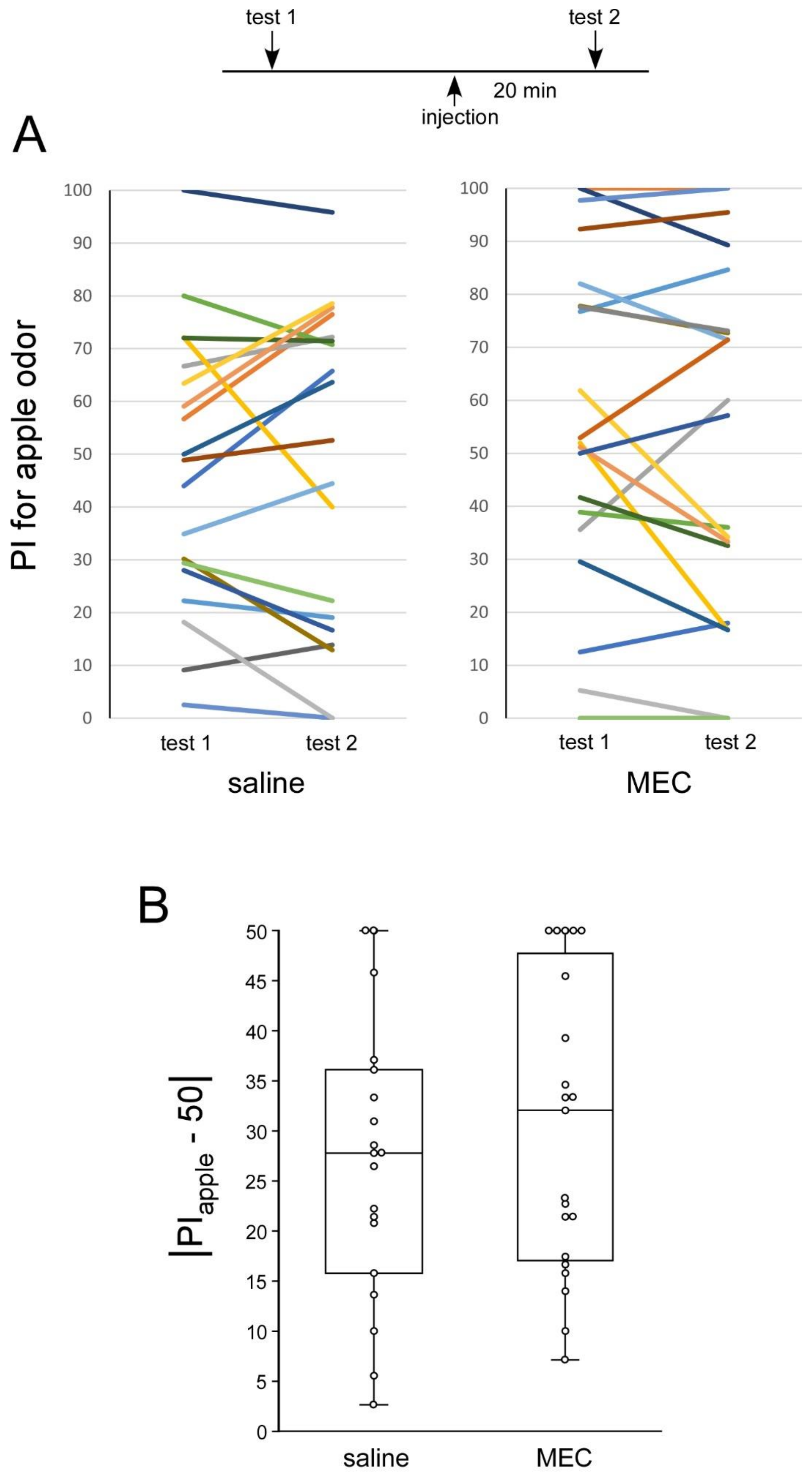

test 1
test 2
injection
20 min
A
PI for apple odor
100
90
80
70
60
50
40
30
20
10
0
test 1
test 2
saline
MEC
B
$|PI_{apple} - 50|$
50
45
40
35
30
25
20
15
10
5
0
saline
MEC

Supplementary Figure S3. Mecamylamine injection did not impair odor discrimination.

The effect of mecamylamine (MEC) injection on odor discrimination was tested. Two groups of crickets were individually injected with 3 μL of saline (control group, n=19) or saline containing 1 mM MEC (n=21). The crickets received odor preference tests in the arena for their odor preference between apple odor and banana odor before and at 20 min after injection. The PI for apple odor (PI_{apple}) was calculated as the percentage of total visiting time to the apple odor source to total visiting time to both odor sources. The PI_{apple} of the control group and MEC-injected group before (test 1) and after (test 2) injection are shown **(A)**. If MEC injection impairs odor discrimination, the bias between the odors would diminish, resulting in PI_{apple} scores of around 50%. However, this was not the case (test 2 in MEC). **(B)** $|PI_{apple} - 50|$ after injection. The absolute difference between PI_{apple} and 50 was calculated to quantitatively analyze the odor bias. If MEC injection impairs odor discrimination, the value of $|PI_{apple} - 50|$ would approach 0. However, the value of $|PI_{apple} - 50|$ in the MEC group (median = 32.1) did not converge to 0 and was not significantly different from the value of $|PI_{apple} - 50|$ in the control group (M-W test, P=0.3935). Thus we concluded that the MEC injection did not impair odor discrimination in this odor pair.
