# Supplementary material for: Critical roles of nicotinic acetylcholine receptors in olfactory memory formation and retrieval in crickets
Source: Front Physiol. 2024 Feb 9;15:1345397. doi: 10.3389/fphys.2024.1345397 (PMC10884312; doi:10.3389/fphys.2024.1345397)
Supplement: Supplementary file 4 [file Image1.pdf]

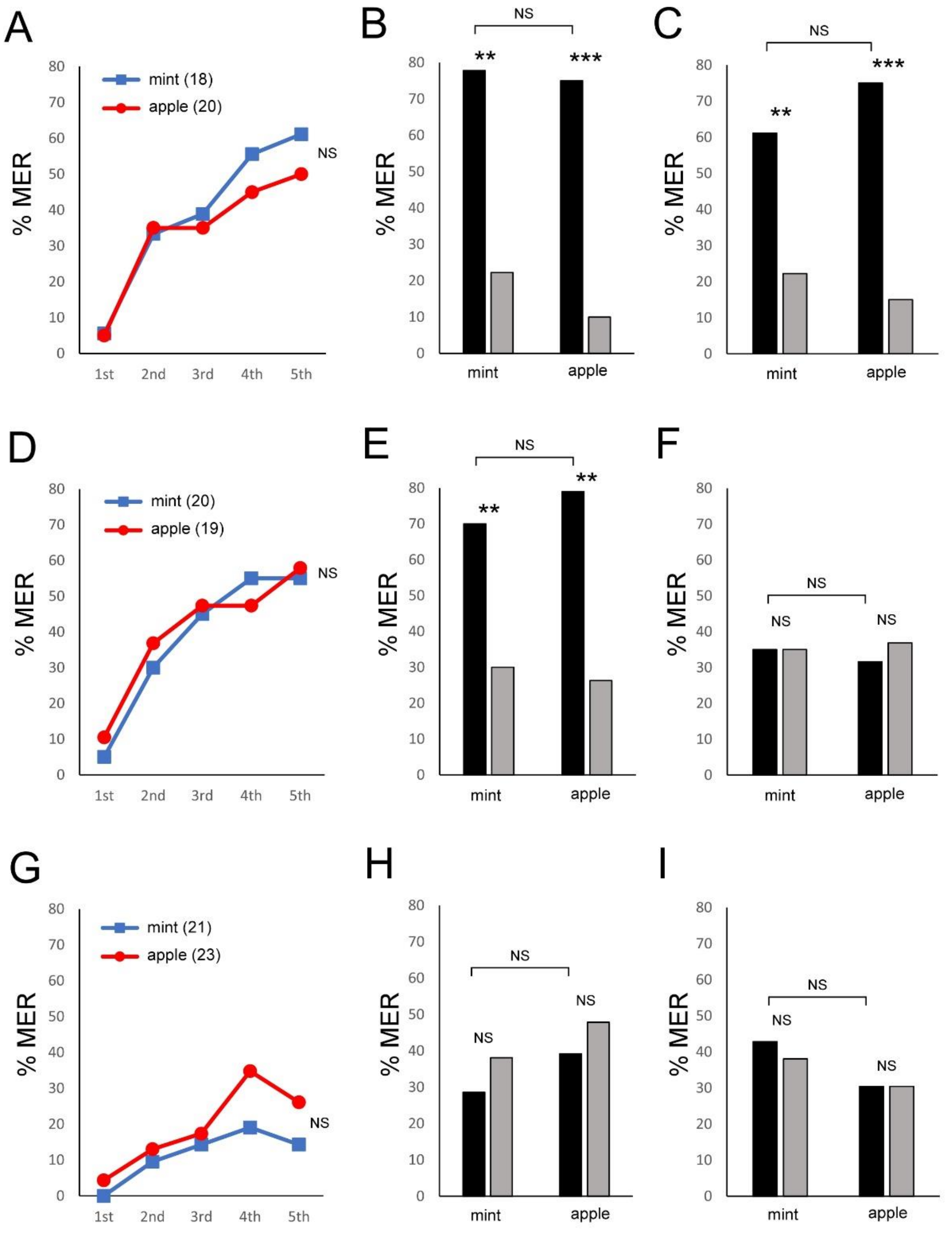

Supplementary Figure S1. No odor bias in appetitive MER conditioning with peppermint CS and apple CS

At 20 min before appetitive conditioning, crickets in three groups were each injected with 3 μL of saline (saline group: **A-C**), saline containing 100 μM MLA (MLA group: **D-F**) or 1 mM MEC (MEC group: **G-I**). (**A, D, G**) Acquisition performance of appetitive

conditioning in the peppermint CS group and the apple CS group. The percentage of MER (%MER) during a 3-sec period of CS (peppermint or apple odor) presentation prior to US (water) presentation is shown. The number of animals tested is shown in parentheses. The %MER to the peppermint odor (blue) or apple odor (red) in the saline group **(A)** was less than 10% in the first trial (i.e., immediately before the first CS-US pairing trial), but it increased to more than 50% in the fifth trial (after the fourth trial). The increase in %MER to the CS with an increase in the number of trials was statistically significant (repeated measures ANOVA, df=4, P=0.0045 in peppermint CS group, P=0.0260 in apple CS group). While the results in the MLA injected group were similar to the control group (repeated measures ANOVA, df=4, P=0.0034 in peppermint CS group, P=0.0328 in apple CS group), the MEC group did not show a significant increase in %MER to the CS with an increase in the number of trials (repeated measures ANOVA, df=4, P=0.3731 in peppermint CS group, P=0.0840 in apple CS group). To add, in all three injection groups, %MER to the CS did not significantly differ between the peppermint CS group and the apple CS group (Fisher's exact test, P>0.05). Thus, data from the two groups were pooled in Figure. 2A. (**B, E, H**) Retention performance at around 10 min after conditioning. In the retention test, each cricket was tested with the CS and the novel odor separated by a 4-min interval. The saline group (**B**) and MLA group (**E**) exhibited a significantly higher %MER to the CS (black bars) than that to the novel odor (gray bars), indicating that the memory is CS-specific. In contrast, in the MEC group (**H**), %MER to the CS was as low as that to the novel odor, indicating no CS-specific short-term memory (STM). (**C, F, I**) Retention performance at 1 day after conditioning. The saline group (**C**) exhibited a significantly higher %MER to the CS (black bar) than that to the novel odor (gray bar), indicating that the memory is CS-specific. In contrast, in the MLA group (**F**)

and the MEC group (**I**), %MER to the CS was as low as that to the novel odor, indicating no CS-specific LTM. Within each of the injection groups (saline, MLA, or MEC), there were no significant differences between the peppermint CS group and the apple CS group (Fisher's exact test, $P>0.05$). Thus, the data from the two CS groups were pooled in Figure. 2B, C. A repeated measures ANOVA was used for within-group comparison of %MER during acquisition. McNemar's test was used for pairwise comparison of %MER between the CS and the novel odor in the retention test. Fisher's exact test was used for pairwise comparison of %MER of different groups in each conditioning trial. The results of statistical comparisons are shown by asterisks (*** $P<0.001$, ** $P<0.01$, NS $P>0.05$).
